# Acute Inflammatory Pseudoaneurysm of the Gastroepiploic Artery in a Patient with Multiple Aneurysms: A Case Highlighting Systemic Vascular Remodeling

**DOI:** 10.3400/avd.cr.25-00072

**Published:** 2025-08-20

**Authors:** Yuri Yoshida, Shinsuke Kikuchi, Daiki Uchida, Naoya Kuriyama, Yuki Tada, Atsuhiro Koya, Sayaka Yuzawa, Hisashi Uchida, Mishie Tanino, Nobuyoshi Azuma

**Affiliations:** 1 Department of Vascular Surgery, Asahikawa Medical University, Asahikawa, Hokkaido, Japan; 2 Department of Cardiovascular Surgery, Sapporo Kosei General Hospital, Sapporo, Hokkaido, Japan; 3 Department of Vascular Surgery, Asahikawa City Hospital, Asahikawa, Hokkaido, Japan; 4 Department of Vascular Surgery, Moriyama Hospital, Asahikawa, Hokkaido, Japan; 5 Department of Cardiovascular Surgery, Hakodate Municipal Hospital, Hakodate, Hokkaido, Japan; 6 Department of Diagnostic Pathology, Asahikawa Medical University Hospital, Asahikawa, Hokkaido, Japan

**Keywords:** gastroepiploic artery aneurysm, visceral artery aneurysm, inflammation

## Abstract

A 52-year-old man with scoliosis and psoriasis vulgaris, treated with infliximab, presented with a large right gastroepiploic artery aneurysm (GEAA). Following surgical resection, additional aneurysms of the anterior communicating artery and abdominal aorta were identified. Histopathological examination revealed a pseudoaneurysm with organizing thrombus and marked acute inflammation, including neutrophilic infiltration of the medial wall. Despite negative cultures, an infection-related vascular insult could not be excluded. This case highlights a rare immune-mediated vascular pathology in the context of chronic inflammatory disease, emphasizing the potential role of acute inflammation and psoriasis-associated immune dysregulation in visceral artery aneurysm formation.

## Introduction

Visceral artery aneurysms (VAAs) are being detected with increasing frequency due to the widespread use of advanced diagnostic imaging modalities.^1)^ Although gastroepiploic artery aneurysms (GEAAs) remain exceedingly rare, the incidence of their identification is expected to rise. While atherosclerosis is a leading cause of VAAs, a variety of other pathogenic mechanisms, including immune-mediated vascular changes and infectious etiologies, may contribute to aneurysm development. In particular, inflammatory remodeling of the vascular wall has emerged as a key process in aneurysmal transformation.^2)^ This case underscores the significance of acute inflammation as a pathogenic mechanism in VAA formation and highlights the presence of multiple aneurysms as indicative of an underlying systemic arterial disorder. Recognition of such presentations is essential, particularly in patients with complex inflammatory or immunosuppressive conditions.

## Case Report

**Figure 1:**
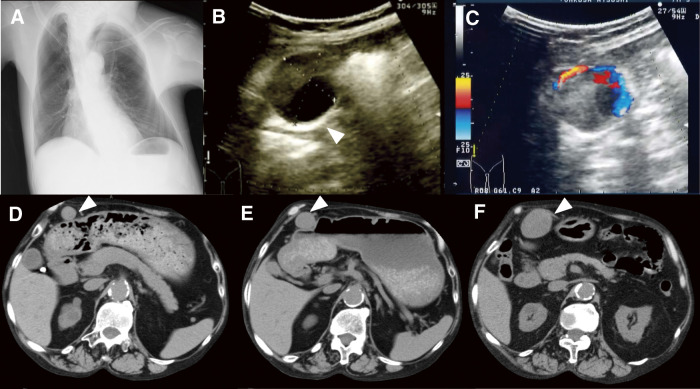
Fig. 1 Image findings. A chest X-ray revealed a scoliosis deformity (**A**). A sagittal sonogram identified a large anechoic mass (arrowhead) in the right hypochondrium (**B**). Color Doppler sonography demonstrated blood flow within the mass (**C**). Computed tomography imaging showed progressive enlargement of the gastroepiploic artery aneurysm, with its maximum diameter measuring 18 mm one year prior (**D**), increasing to 26 mm five months before admission (**E**), and expanding to 50 mm at the time of admission (**F**).

A 52-year-old man with a history of psoriasis vulgaris underwent routine abdominal ultrasound at another hospital during a health check. The patient had a long-standing diagnosis of psoriasis vulgaris, managed with biologic agents. Treatment initially involved infliximab (300 mg/day), administered at intervals ranging from 1 to 2 months over several years. Subsequently, ustekinumab (90 mg/day) was introduced and continued every 2–3 months. The examination incidentally revealed a 3.5 cm circular anechogenic area in the right hypochondrium, suggestive of an intra-abdominal mass. He was subsequently referred to our hospital for further evaluation. The patient was asymptomatic and had no history of abdominal trauma, peptic ulcer disease, or pancreatitis. His medical history included scoliosis (**Fig. 1A**), diabetes mellitus, hypertension, and end-stage renal disease (ESRD) without dialysis, with a serum creatinine level of 4.77 mg/dL. His ongoing treatment consisted of hydrocortisone, which was administered at a dosage of 20 mg/day to manage adrenal insufficiency associated with ESRD, as well as β-blockers and calcium-channel blockers. Hydrocortisone therapy was initiated approximately 6 months before the GEAA was resected. Laboratory tests showed a C-reactive protein level of 0.21 mg/dL. Although comprehensive autoimmune serology was not performed at the time of the initial presentation, the patient’s erythrocyte sedimentation rate (ESR) was elevated at 24 mm/hour, suggesting a mild degree of systemic inflammation. Ultrasound examination detected an arterial pulse within the mass, suggesting an aneurysm (**Figs. 1B** and **1C**). A review of previous computed tomography (CT) scans, routinely performed to monitor the tuberculosis risk associated with infliximab therapy, revealed progressive enlargement of the aneurysm from 26 to 50 mm over 5 months (**Figs. 1D**–**1F**). Based on the ultrasound and CT findings, the mass was diagnosed as a right GEAA. Given the aneurysm’s size and location, as well as the patient’s ESRD, which limited the feasibility of contrast-enhanced endovascular therapy, open surgical resection was chosen as the preferred treatment approach.

**Figure 2:**
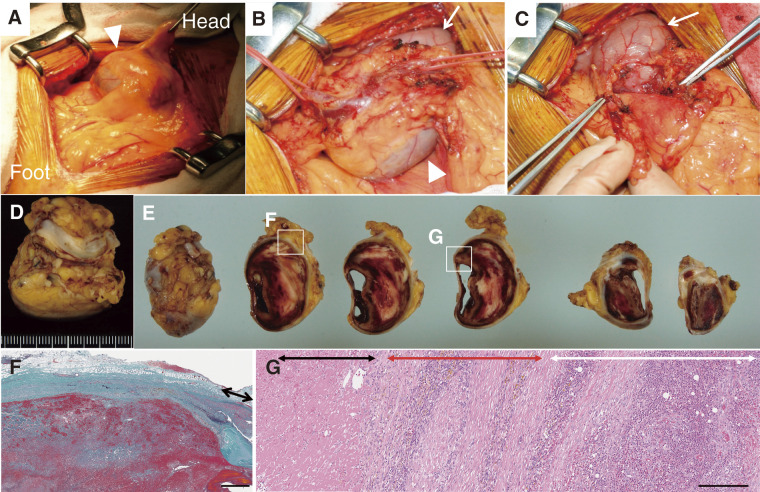
Fig. 2 Intraoperative and histological findings. The aneurysm was located beneath the peritoneum (**A**), with the inflow and outflow arteries of the GEAA, along with the accompanying veins, carefully freed and taped (**B**). Following aneurysm resection (**C**), most of the aneurysmal sac was found to be filled with thrombus, although a portion remained patent (**D**, **E**). Arrowheads in (**A**) and (**B**) indicate the GEAA, while arrows in (**B**) and (**C**) indicate the stomach. Histopathological examination revealed a focal area of the aneurysmal wall retaining a normal 3-layer structure (black arrow, **F**); however, the majority of the wall was entirely replaced by granulation tissue (**F**; red arrow, **G**). The aneurysm lumen was filled with organizing thrombus (white arrow, **G**). Focal abscess formation was observed in the adventitia (white arrow, **G**), although no bacteria or fungi were identified. (**F**) Elastica-Masson staining. (**G**) Hematoxylin and eosin staining. Scale bars: (**F**) 1 mm; (**G**) 250 μm. GEAA: gastroepiploic artery aneurysm

A midline incision provided access to the peritoneal cavity, where the aneurysm was readily identified distal to the stomach (**Fig. 2A**). The proximal and distal ends of the right gastroepiploic artery were ligated, and the aneurysm was resected after being freed from the stomach and omentum (**Figs. 2B** and **2C**). The aneurysm was predominantly filled with thrombus, though partial luminal patency was observed (**Figs. 2D** and **2E**). Histopathological examination demonstrated that granulation tissue without a normal 3-layer structure replaced most of the aneurysm wall. The aortic lumen was filled with organizing thrombus composed of red blood cells, fibrin, neutrophils, nuclear debris, and focal fibroblasts. Where the thrombus was attached, neutrophils also infiltrated the medial portion of the aneurysmal wall, accompanied by lymphoplasmacytic infiltration, capillary neovascularization, and hemosiderin deposition. Additionally, an abscess was present in the adventitia of the aneurysmal wall. However, Gram and Grocott staining revealed no bacteria or fungi, respectively, and bacterial cultures were negative (**Figs. 2F** and **2G**). There were no multinucleated giant cells or fibrinoid necrosis of the vessel wall. The aneurysm was classified as a pseudoaneurysm with acute inflammation.

**Figure 3:**
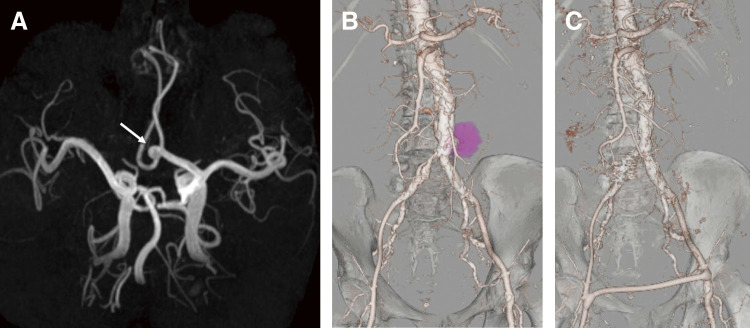
Fig. 3 Multiple aneurysm formation after resection of a gastroepiploic artery aneurysm. An anterior communicating artery aneurysm was incidentally discovered (arrow, **A**). A saccular abdominal aortic aneurysm subsequently developed (**B**). Endovascular aortic repair was performed, including right common iliac artery embolization and femoro-femoral bypass grafting (**C**).

The patient’s postoperative course was uneventful, with no wound-related complications or deterioration of renal function. However, 17 months after surgery, his ESRD progressed, necessitating the initiation of hemodialysis. Subsequently, 18 months after GEAA resection, an anterior communicating artery aneurysm (AComAA) was treated with coil embolization (**Fig. 3A**). Regarding differentiation from infectious endocarditis as a potential cause of AComAA formation, transthoracic echocardiography revealed no valvular abnormalities, vegetations, or intracardiac masses. Additionally, no systemic signs suggestive of endocarditis, such as embolic events or constitutional symptoms, were observed. A saccular abdominal aortic aneurysm (AAA) was managed with endovascular aortic repair 25 months postoperatively (**Fig. 3B**). Given severe calcification and significant stenosis of the right common iliac artery (CIA), along with the small diameter of the abdominal aorta, additional interventions, including right CIA embolization and femoro-femoral artery bypass grafting, were required to optimize vascular flow (**Fig. 3C**). The postoperative course following EVAR was uneventful, and the patient was discharged without any procedure-related complications. Aside from routine perioperative prophylaxis during GEAA resection and EVAR, no antibiotics were administered throughout the broader clinical course, as there was no clinical indication for infection before, between, or after these procedures. Unfortunately, 37 months after GEAA resection, the patient suffered a sudden death, likely attributable to underlying vascular pathology and progressive renal disease.

## Discussion

VAAs are rare vascular abnormalities typically regarded as structurally stable lesions associated with atherosclerosis or degenerative changes. However, an increasing body of evidence suggests that systemic factors, including immune dysregulation and inflammation, may contribute to their development and progression.^2)^ Traditionally, GEAA was diagnosed in patients presenting with hemoperitoneum, but advancements in imaging modalities such as CT, ultrasound, and magnetic resonance imaging have improved detection rates for unruptured VAAs.^2,3)^ In the present case, a GEAA was incidentally detected and subsequently resected. Management of VAAs is guided by various factors, including pseudoaneurysm formation, symptomatic presentation, rupture risk, and aneurysm diameter exceeding 2.5 cm.^4)^ Although GEAA is an exceedingly rare subtype of VAA,^1)^ its clinical significance lies in its high rupture risk, which exceeds 80% regardless of aneurysm size.^3,5)^ Reflecting this risk, the Society for Vascular Surgery recommends treatment for all gastric artery aneurysm and GEAA, irrespective of their dimensions.^2)^ Prompt diagnosis and timely intervention are essential in the management of VAAs, particularly given their potential for rupture. While endovascular embolization is widely regarded as the 1st-line treatment, especially in emergency settings,^2)^ it carries risks such as distal thromboembolism, nontarget vessel occlusion, coil migration, end-organ infarction, and intraprocedural rupture.^4)^ Additionally, aneurysms larger than 2 cm are more prone to coil compaction or recanalization, often necessitating re-intervention.^6)^ In anatomically favorable cases, surgical resection remains a definitive alternative. In the present case, open surgical excision was selected due to the aneurysm’s size, renal dysfunction, and vascular complexity. The subperitoneal location of the GEAA allowed for direct visualization and precise resection during laparotomy (**Fig. 2A**).

Histopathological examination revealed prominent acute inflammation within the aneurysmal wall, including dense neutrophilic infiltration and disruption of the elastic lamina. Although Gram and Grocott staining were negative and culture results remained sterile, the possibility of an infectious etiology could not be definitively excluded, given the patient's immunosuppressive state due to systemic therapy for psoriasis vulgaris. Immunosuppression may obscure typical clinical and microbiological signs of infection, particularly in the setting of localized vascular pathology. Therefore, the observed findings are suggestive of a possible infection-related vascular injury, despite the absence of direct microbiological confirmation. Segmental arterial mediolysis (SAM) has also been described as a cause of visceral aneurysms.^2)^ However, its characteristic histologic features, such as media lysis without inflammation, were not observed. In contrast, this case showed pronounced immune cell infiltration, granulation tissue formation, and abscess development, effectively ruling out SAM as the primary etiology.

The presence of multiple aneurysms, including an AComAA and a saccular AAA, suggests an underlying systemic arterial pathology. Although connective tissue disorders such as Ehlers–Danlos syndrome, arterial tortuosity syndrome, and Loeys–Dietz syndrome are known to predispose to aneurysm formation,^7–9)^ no definitive genetic or histopathologic evidence supported these diagnoses in this case. Instead, histological analysis revealed marked acute inflammation within the aneurysmal wall, indicating a rapidly destructive vascular process. Systemic vasculitis was considered among the differential diagnoses, including conditions such as Behçet’s disease and anti-neutrophil cytoplasmic antibody (ANCA)-associated vasculitis. However, the clinical presentation lacked features suggestive of Behçet’s disease, such as oral or genital ulcers, uveitis, or characteristic skin lesions. In addition, histopathological analysis revealed no evidence of granulomatous inflammation or fibrinoid necrosis. Nonetheless, we acknowledge that autoimmune serologic testing, including ANCA, was not comprehensively performed in this case. Therefore, the possibility of an underlying vascular inflammatory disease cannot be entirely excluded. While chronic immune-mediated remodeling is well recognized in aneurysm pathogenesis, this case highlights the potential for acute immune activation, possibly involving interleukin-6 and tumor necrosis factor-α (TNF-α), as well as a mildly elevated ESR, to drive abrupt vascular degradation.^10)^ The patient had a history of treatment with infliximab and later ustekinumab for psoriasis vulgaris. Both agents exert anti-inflammatory effects by targeting TNF-α and interleukin-12/interleukin-23 signaling pathways, respectively. While they are expected to suppress systemic inflammation and may potentially reduce vascular remodeling, their immunosuppressive properties may also impair local host defense, allowing for subclinical infection or exaggerated tissue injury. In the present case, despite negative cultures, histopathology revealed abscess formation in the adventitia and neutrophilic infiltration, suggesting an infectious or immune-mediated vascular insult. We interpret the use of biologics, particularly TNF-α blockade, as a double-edged factor: attenuating chronic inflammation but predisposing to occult infection and impaired vascular healing, potentially contributing to pseudoaneurysm formation. Supporting this, a nationwide Danish cohort study reported a significantly increased risk of AAAs among patients with severe psoriasis, underscoring the vascular consequences of systemic inflammation. Even in the absence of confirmed infection, such inflammatory insults may compromise endothelial integrity and medial structure, ultimately contributing to pseudoaneurysm formation and destabilization. From a treatment perspective, given the presence of histological abscess formation and the use of immunosuppressive therapy, the potential role of occult infection in pseudoaneurysm development warrants careful consideration. Although no clinical or microbiological evidence supported an infectious etiology in the present case, including negative blood and intraoperative cultures, the possibility of subclinical infection cannot be entirely excluded. In this context, empirical or prophylactic antibiotic administration may merit discussion as part of the therapeutic strategy, especially in immunocompromised patients presenting with acute aneurysmal inflammation.

In summary, this case illustrates a multifactorial pathogenesis for VAA formation involving acute arterial inflammation, possible occult infection, systemic immune dysregulation, and chronic immunosuppression. Recognition of such mechanisms is vital for guiding diagnostic and therapeutic strategies, especially in patients presenting with multiple aneurysms and underlying inflammatory disease. Further investigation is needed to elucidate the contributions of immune-mediated vascular remodeling in aneurysm pathophysiology and to determine appropriate surveillance protocols for affected individuals.

## Conclusion

This case highlights a rare, acutely inflamed visceral pseudoaneurysm likely driven by immune dysregulation. The presence of multiple aneurysms suggests systemic vascular vulnerability, warranting long-term monitoring and further investigation into inflammation-mediated vascular pathology.

## Declarations

### Informed consent

The patient provided written consent at the time of discharge for his information to be used for research and publication.

### Disclosure statement

All authors have no conflict of interest.

### Author contributions

Treatment strategy: NA

Data collection: YY, SK, DU, AK

Surgical procedure: YY, SK, DU, AK, NA

Pathological examination: SY, MT

Manuscript preparation: YY, SK, SY

Critical review and revision: all authors

Final approval of the article: all authors

Accountability for all aspects of the work: all authors.
